# Molecular characteristics and physiological roles of Na^+^–K^+^–Cl^−^ cotransporter 2

**DOI:** 10.1002/jcp.29997

**Published:** 2020-08-10

**Authors:** Andree‐Anne Marcoux, Laurence E. Tremblay, Samira Slimani, Marie‐Jeanne Fiola, Fabrice Mac‐Way, Alexandre P. Garneau, Paul Isenring

**Affiliations:** ^1^ Department of Medicine, Nephrology Research Group Laval University Quebec City Québec Canada; ^2^ Cardiometabolic Axis, School of Kinesiology and Physical Activity Sciences University of Montréal Montréal Quebec Canada

**Keywords:** Bartter syndrome, cation‐Cl^−^ cotransporter, high blood pressure, loop of Henle, Na^+^–K^+^–Cl^−^ cotransporter 2, renal physiology

## Abstract

Na^+^–K^+^–Cl^−^ cotransporter 2 (NKCC2; SLC12A1) is an integral membrane protein that comes as three splice variants and mediates the cotranslocation of Na^+^, K^+^, and Cl^−^ ions through the apical membrane of the thick ascending loop of Henle (TALH). In doing so, and through the involvement of other ion transport systems, it allows this nephron segment to reclaim a large fraction of the ultrafiltered Na^+^, Cl^−^, Ca^2+^, Mg^2^^+^, and HCO_3_^−^ loads. The functional relevance of NKCC2 in human is illustrated by the many abnormalities that result from the inactivation of this transport system through the use of loop diuretics or in the setting of inherited disorders. The following presentation aims at discussing the physiological roles and molecular characteristics of Na^+^–K^+^–Cl^−^ cotransport in the TALH and those of the individual NKCC2 splice variants more specifically. Many of the historical and recent data that have emerged from the experiments conducted will be outlined and their larger meaning will also be placed into perspective with the aid of various hypotheses.

## Graphical abstract

Na^+^–K^+^–Cl^−^ cotransporter 2 (NKCC2) mediates the cotranslocation of Na^+^, K^+^, and Cl^−^ ions through the apical membrane of the thick ascending loop of Henle (TALH). In doing so, it allows this nephron segment to reclaim a large fraction of the ultrafiltered Na^+^, Cl^−^, Ca^2+^, Mg^2^^+^, and HCO_3_^−^ load. The functional relevance of NKCC2 in humans is illustrated by the many abnormalities that result from the inactivation of this transport system.

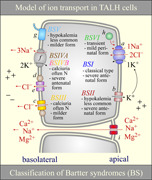

## 1 INTRODUCTION

Na^+^–K^+^–Cl^−^ cotransporter 2 (NKCC2; SLC12A1) is a cation‐Cl^−^ cotransporter (CCC) that shares high homology in residue sequence with two of its family members, that is, with NKCC1 (SLC12A2) and Na^+^–Cl^−^ cotransporter (NCC; SLC12A3). Its main role is to mediate the cotranslocation of Na^+^, K^+^, and Cl^−^ through the apical pole of the thick ascending loop of Henle (TALH) in the mammalian kidney (Kaplan et al., 1996; Nielsen, Maunsbach, Ecelbarger, & Knepper, 1998). Because of this transport mechanism, NKCC2‐expressing cells can reclaim up to 25% of the ultrafiltered NaCl load and sustain the reabsorption of other solutes, such as Ca^2+^, Mg^2^^+^, and HCO_3_^−^. In the distal TALH, they are also involved in a process known as tubuloglomerular feedback (TGF). NKCC2 is expressed in a number of extrarenal tissues as well but at lower levels.

NKCC2 was cloned just before the mid‐1990s by two different research groups. It was initially found to come as three main splice variants that exhibit different functional traits, distributions, and physiological roles along the TALH (Gamba et al., 1994; Payne & Forbush, 1994). After its discovery, NKCC2 was linked to an antenatal form of salt‐losing nephropathy called classical Bartter syndrome and to blood pressure (BP) variations in the population (Acuna et al., 2011; Simon et al., 1996; Starremans, Kersten, Knoers, van den Heuvel, & Bindels, 2003). In the TALH, other ion transport systems that provide NKCC2 with the required driving force to sustain the coupled uptake of ions have also been linked to disorders of renal salt handling.

The following review aims at presenting and discussing the physiological roles and molecular characteristics of the NKCC2 splice variants along the TALH and in extrarenal cell types. As will be seen, much progress has been made in these regards over the last 40 years. However, many challenges still need to be overcome for new and potentially exciting developments to take place in the field of renal Na^+^–1K^+^–Cl^−^ cotransport. Such challenges include the development of protein probes and pharmacological inhibitors that are specific to each of the splice variants. A recent publication on the high‐resolution structure of NKCC1 should become quite helpful to this end.

## 2 MAIN TEXT

### 2.1 Characterization of Na^+^–1K^+^–Cl^−^ cotransport in the TALH before the molecular era

#### 2.1.1 Early identification and localization

In the late 1970s, an electrically silent furosemide‐sensitive 1Na^+^–1K^+^–2Cl^−^ cotransport mechanism was identified in Ehrlich–Lettre ascites carcinoma cells by Geck, Pietrzyk, Burckhardt, Pfeiffer, and Heinz (1980) and reported to differ from the Na^+^/K^+^‐ATPase pump in many aspects. For instance, this mechanism was found to be ouabain‐insensitive, to allow the movement of ions in the absence of ATP hydrolysis, and to promote cell swelling. It is now known to be mediated by NKCC1 or SLC12A2 and to be widely distributed. Ion translocation by this transport system was later characterized in duck red blood cells and reported to be ordered according to a model of glide symmetry (Lytle, McManus, & Haas, 1998).

Soon after the discovery of NKCC1, a furosemide‐sensitive Na^+^–1K^+^–Cl^−^ cotransport mechanism in the TALH was formally identified by Greger and Schlatter (1981) through renal tubule microperfusion studies and shown to be functionally similar to the cotransport mechanism of Ehrlich–Lettre cells. Importantly, an active Cl^−^ transport system had already been uncovered on the luminal side of the TALH in the early 1970s and found to be the primary target of furosemide or other high ceiling diuretics (Burg, Stoner, Cardinal, & Green, 1973; Rocha & Kokko, 1973). However, its requirement for K^+^ had not been demonstrated during that time. In the TALH, this transport system is now known to be mediated by NKCC2 or SLC12A1.

During its initial characterization in the kidney, the K^+^‐dependent Cl^−^ cotransport mechanism was found to be deployed along the entire TALH and in the macula densa (MD) but to operate at higher capacity in the medulla (Greger & Schlatter, 1981; Koenig, Ricapito, & Kinne, 1983). In the early 1970s, interestingly, rates of luminal fluid dilution by the TALH had also been found to be higher in the cortex (Burg, 1982; Burg & Green, 1973; Greger & Schlatter, 1981; Koenig et al., 1983; Rocha & Kokko, 1973). These findings were, thus, already consistent with the existence of different forms of NKCCs in the kidney, that is, with a cotransporter of high capacity in the medullary TALH and a cotransporter of high affinity in the cortical TALH.

#### 2.1.2 Stoichiometry of ion transport

The stoichiometry of the renal Na^+^–1K^+^–Cl^−^ cotransport mechanism was characterized more precisely in the mid‐1980s through ^86^Rb influx studies in ion‐free membrane vesicles isolated from the apical domain of LLCPK1 cells (Brown & Murer, 1985). The kinetic parameters obtained—a Hill coefficient of 1.63 for Cl^−^ transport and *V*_max_ of similar magnitudes for Na^+^ and K^+^ transport—were also consistent with a 1Na^+^:1K^+^:2Cl^−^ coupling mode. Noticeably, LLCPK1 cells had not been treated before the measurements to have their cAMP_i_ levels rise above background levels (see Section 2.3.2). Otherwise, no studies have been carried out thus far to determine whether ion binding by the renal cotransporter was ordered.

#### 2.1.3 Regulation

During the 15 years that followed the identification of a K^+^‐dependent Cl^−^ transport system in the TALH, further renal tubule microperfusion studies allowed to gain key insight into the mechanisms of carrier regulation. In particular, vasopressin and calcitonin were found to exert a stimulatory effect on ion cotransport by increasing cAMP_i_, and PGE2 to exert the opposite effect (Di Stefano et al., 1990; Dublineau, Pradelles, de Rouffignac, & Elalouf, 1992; Torikai & Kurokawa, 1983; Wittner, Di Stefano, Mandon, Roinel, & de Rouffignac, 1991). In one study, vasopressin was also shown to change the stoichiometry of ion cotransport from a 1Na^+^:0K:1Cl^−^ to 1Na^+^:1K^+^:2Cl^−^ coupling mode (Sun, Grossman, Lombardi, & Hebert, 1991). As for the potential cAMP‐dependent downstream effectors at play, F‐actin was identified as one of the candidates based on transport assays in primary cultures of mouse (ms) TALH cells (Wu, Bens, Cluzeaud, & Vandewalle, 1994).

#### 2.1.4 Evidence for a new isoform

From the early‐1980s to mid‐1990s, many investigators felt that the protein responsible for Na^+^–1K^+^–Cl^−^ cotransport in the TALH was not the same as the protein responsible for Na^+^–1K^+^–Cl^−^ cotransport in extrarenal tissues. Even if this possibility was suggested by a number of indirect observations, there was no evidence to the contrary. Moreover, both the renal and extrarenal transport mechanisms could have differed in functional properties because of cell‐specific factors.

### 2.2 Identification of NKCC2 and of several NKCC2 splice variants

#### 2.2.1 Cloning of NKCC2

The residue sequence of the TALH cotransporter was identified in the mid‐1990s and found to differ from that of the extrarenal cotransporter as suspected. It was uncovered more specifically by two independent research groups, that is, by Payne and Forbush(1994) who termed the protein NKCC2—as they had discovered the sequence of the extrarenal isoform just before—and by Gamba et al. (1994) who termed it bumetanide‐sensitive cotransporter type 1 (BSC1)—as they had not yet discovered the sequence of the extrarenal isoform. As it stands, the terms BSC1 and BSC2, which refer to NKCC2 and NKCC1, respectively, have been largely abandoned.

For Payne and Forbush (1994), the identification strategy was based on the premise that the renal and ubiquitous carriers would share similar residue sequences as they shared similar transport functions. By probing an RNA blot of *Squalus acanthias* tissues with a radiolabeled NKCC1‐specific complementary RNA (cRNA) in previous studies, Xu et al. (1994) had also identified an RNA transcript of a smaller size in the kidney. Two rabbit (rb) kidney complementary DNA (cDNA) libraries (one from the medulla and one from cortex) were thus screened at low stringency with an rbNKCC1‐derived probe and found to include the sequences of interest. These experiments had also allowed revealing that NKCC2 came as three main splice variants that were then called NKCC2B, NKCC2A, and NKCC2F.

For Gamba et al. (1993), the identification strategy was based on similar premises except that the sequence used as a query consisted of the NCC. This other carrier had been cloned from *Pseudopleuronectes americanus* by the same research group a year before and was also known to share similar transport functions with the extrarenal NKCC. A rat (rt) kidney size‐selected cDNA library from the outer medullary inner stripe (OMIS) was thus screened with rtNCC‐derived radioactive probes and found to include the sequences of interest. However, the only variant identified in this library was NKCC2F.

**Figure 1:**
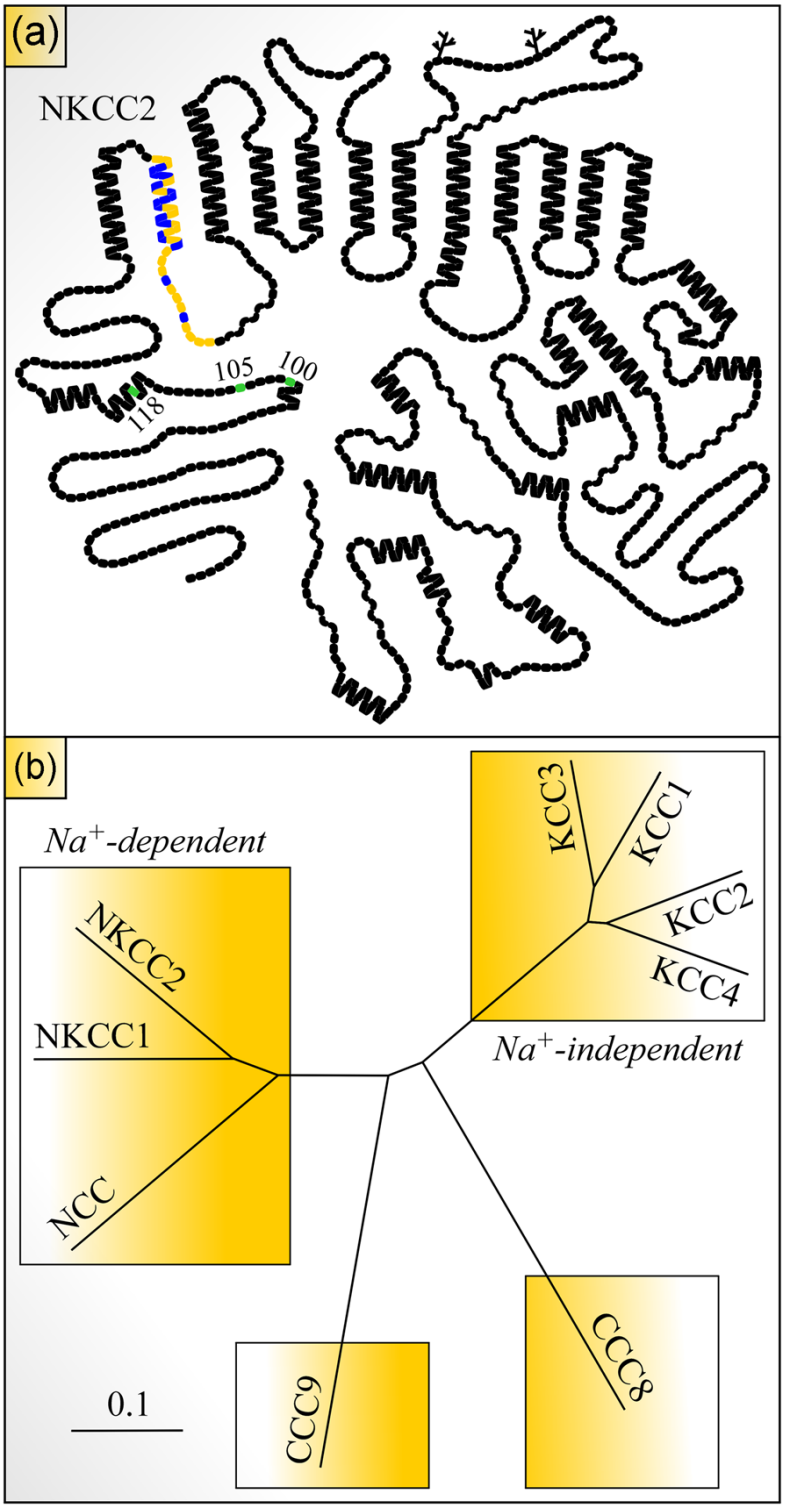
Hydropathy plot model of NKCC2 and phylogram of CCC family. (a) Residues are shown as round or square forms (one form per residue) and putative glycosylation sites as branched lines. Residues in yellow are those of the alternatively spliced exon, residue in blue differ among the variants, and residues in green correspond to known phosphoacceptor sites in the N‐terminus. The model was drawn using the program PLOT. (b) The tree was constructed through the programs Clustal Omega and FigTree v1.4.3 (Sievers et al., 2011) using the longest human (hu) residue sequences for each of the CCCs shown. Members of this family belong to four different subclasses. The scale corresponds to a genetic distance. Sequences used: NKCC1, NP_001037.1; NKCC2, NP_000329.2; NCC, NP_000330.2; KCC1, NP_005063.1; KCC2, NP 001128243.1; KCC3, NP_598408.1; KCC4, NP_006589.2; CCC8, NP_064631.2; CCC9, NP_078904.3. CCC, cation‐Cl^−^ cotransporter; KCC1, K^+^–Cl^−^ cotransporter 1; NCC, Na^+^–Cl^−^ cotransporter; NKCC2, Na^+^–K^+^–Cl^−^ cotransporter 2

The open‐reading frame of both rbNKCC2 and rtNKCC2 was eventually predicted to code for a 1,095‐amino acid protein that forms a central core of 12 transmembrane domains (TMD) flanked by cytosolically disposed extremities (Figure 1a). The translation product was also found to share over 60% identity with NKCC1 and over 50% with NCC with maximal levels of conservation over the membrane‐associated domain. Western blot analyses further revealed that NKCC2 migrated at a ∼160‐kDa landmark and was thus likely to consist of a glycoprotein.

In their initial publication on the cloning of NKCC2, Payne and Forbush (1994) demonstrated that the rbNKCC2A variant acted as a heterologous bumetanide‐sensitive ^86^Rb^+^‐transport carrier system in HEK‐293 cells. However, protein expression at the surface of these cells could only be achieved through the use of a chimera in which the first 104 N‐terminal residues of NKCC2A were replaced by those of human (hu) NKCC1. Since then, it appears that most investigators have failed to obtain heterologous expression of a functional wildtype NKCC2 in extrarenal mammalian cells. To be functional, the renal isoform must presumably interact in most species with a TALH‐specific accessory protein through its proximal N‐terminus.

As for Gamba et al. (1994), the initial characterization of ms NKCC2F was achieved in *Xenopus laevis* oocytes, an amphibian expression system that proved invaluable in the following years to characterize the various properties of the individual splice variants. The data obtained by this group showed that the sequence identified in the rt library did code for an NKCC. In particular, ^86^Rb^+^ influx in NKCC2F‐expressing oocytes was seen to be clearly above background under isotonic condition but not when the extracellular medium was added with bumetanide in the extracellular medium or devoid of Na^+^ or Cl^−^ ions. NKCC2F‐mediated ^86^Rb^+^ transport was also seen to be metolazone‐ and DIDS‐insensitive.

#### 2.2.2 Splice variants

**Figure 2:**
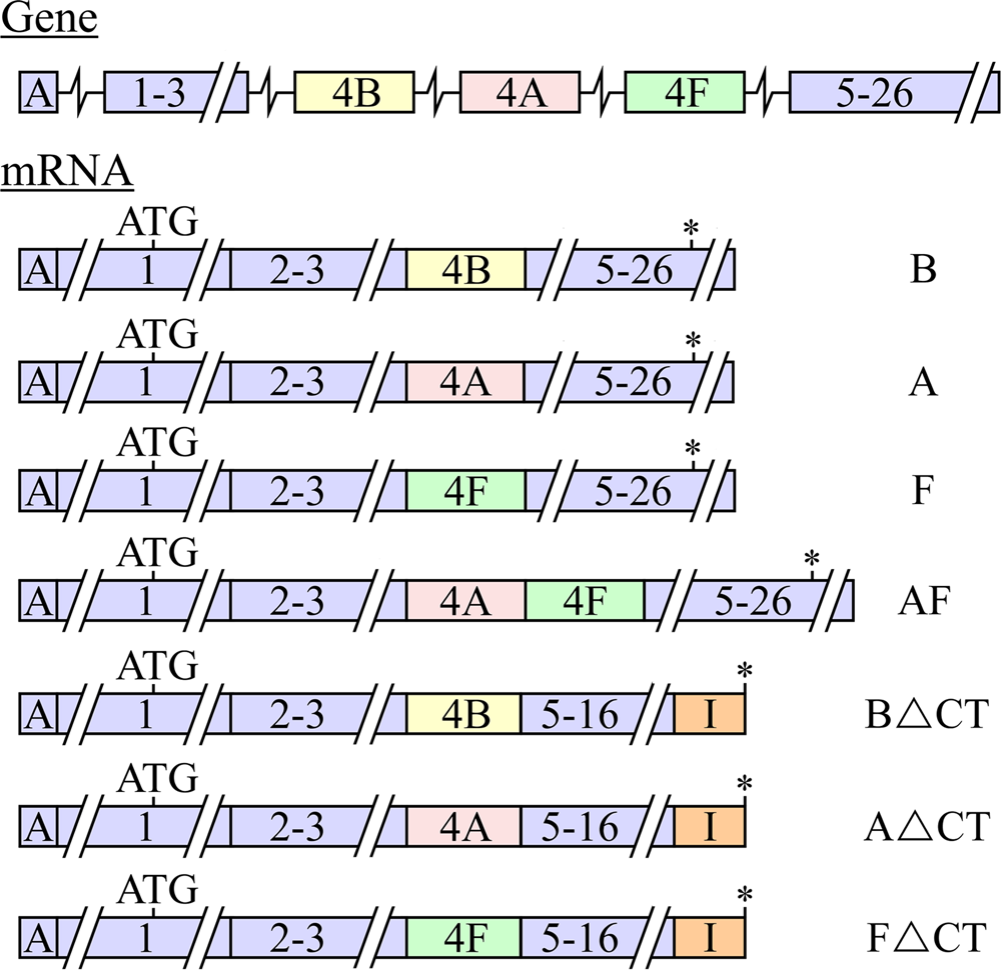
NKCC2 splice variants. The primary transcript counts 97,776 nucleotides from the start of exon A (before exon 1) to the end of exon 26. It is represented in this figure by boxes (for the exons) and lines (for the untranslated regions) that are drawn to scale on the horizontal axis. Regions in orange correspond to neosequences before a polyadenylation site in intron 16. Because of this polyadenylation site, the NKCC2∆CT transcripts should thus form typical polyA‐tailed mRNA. Variants NKCC2B, NKCC2A, and NKCC2F are found in several species such as hu, rabbit (rb), and mouse (ms) kidney while variants NKCC2B∆CT, NKCC2A∆CT, and NKCC2F∆CT are only found in ms kidney (Mount, Baekgaard et al., 1999). I, residues encoded from intronic sequences; mRNA, messenger RNA; NKCC2, Na^+^–K^+^–Cl^−^ cotransporter 2; ∆CT, variants in which exons 17–26 (corresponding to most of the C‐terminus) are missing; *, stop codon

The rbNKCC2 splice variants identified were seen to be produced from the alternative usage of a single exon among three different ones in the primary transcript (Payne & Forbush, 1994). These exons (called 4B, 4A, and 4F) occur in that order from 5′ to 3′, share substantial homology in residue sequence among each other, and are all flanked by intronic sequences. As such, they probably arose during evolution through replication slippage. Importantly, they each code for a 32‐residue segment that forms the second TMD2 and part of the following connecting loop (CL1) of the transporter. The exon–intron organization of the encoding gene is illustrated through Figure 2.

**Figure 3:**
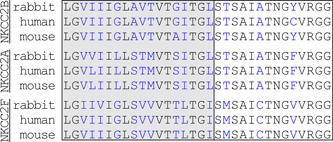
Residue sequences of exon 4 in Na^+^–K^+^–Cl^−^ cotransporter 2 (NKCC2). Blue is used to indicate that residues differ among variants (NKCC2B, NKCC2A, and NKCC2F) and species (rb, hu, and ms). Residues in the gray box belong to transmembrane domain 2 and residues in the white box to composite site 1

An alignment of the TMD2–CL1 domains of NKCC2B, NKCC2A, and NKCC2F in rt, ms, rb, and hu is shown in Figure 3. As can be observed, the TMD2 segment per se is predicted to have 18 residues of which 8 are identical among variants and species. Those that differ (at Positions 213–215, 217, 219–221, 224, 225, and 228 in the figure) are still highly conserved based on Grantham scores. As for CL1, it is predicted to have 14 residues of which 11 are identical among variants and species. Those that differ (at Positions 230, 234, and 238) are also highly conserved. As will be discussed later, the TMD2–CL1 domain of NKCC2 has now been found to play a key role in ion transport and carrier expression at the cell surface.

NKCC2 can also be processed into a mature transcript that encloses both exons 4A and 4F in tandem with no disruption of the open‐reading frame. In rb kidney, this transcript (NKCC2AF) was found to account for ∼3% of the variants produced such that it was considered to be a splicing artifact (Payne & Forbush, 1994). However, it was found to account for as much as ∼30% of the variants in *Squalus acanthias* kidney, and as will be discussed further below, to exert a dominant‐negative effect on the other variants in *X. laevis* oocytes (Brunet et al., 2005; Gagnon et al., 2002). For these reasons, the role of NKCC2AF in the TALH should be viewed as uncertain.

Additional splice variants can also be produced in ms through partial retention of intron 16 and usage of an alternative polyadenylation site in this noncoding nucleotide segment (Mount, Baekgaard et al., 1999). The resulting protein contains a unique 55‐residue stretch from the retained intronic sequence, is devoid of exons 17–26 (that codes for most of the intracellular C‐terminus), and can contain either of exons 4B, 4A, and 4F. As will be discussed below once again, Mount, Mercado et al. (1999) and Plata et al. (2001) have shown that these variants (called NKCC2∆CT henceforth) could also play a physiological role in vivo by exerting a cAMP_i_‐dependent dominant‐negative effect on the WT variants in *X. laevis* oocytes.

#### 2.2.3 CCC family

The residue sequences of NKCC1, NKCC2, and NCC were uncovered at about the same time in the early 1990s. They were also predicted to share homology with another protein that was known to act as an electroneutral Na^+^‐independent K^+^–Cl^−^ cotransporter. When this protein was identified in the late 1990s, it was not only found to bear similitude with the three other ones, but also to come as four isoforms that were called K^+^–Cl^−^ cotransporter 1 (KCC1), KCC2, KCC3, and KCC4 (Gillen, Brill, Payne, & Forbush, 1996; Hiki et al., 1999; Mount, Mercado et al., 1999; Payne, Stevenson, & Donaldson, 1996; Race et al., 1999). The term CCC is now commonly used as the family name under which the NKCCs, NCC, and the KCCs are regrouped.

As it stands, nine CCCs have now been uncovered (Garneau et al., 2017; Garneau, Marcoux et al., 2019; Garneau, Slimani et al., 2019; Marcoux et al., 2017). As shown in Figure 1b, they fall into four phylogenetic clades. NKCC2 belongs to one of these clades along with NKCC1 and NCC, that is, to the Na^+^‐dependent CCC clade, the KCCs belong to the Na^+^‐independent CCC clade and the two remaining CCCs (CCC8 and CCC9) each belong to an individual clade. Overall, the Na^+^‐dependent CCCs share ∼50% identity in amino acid sequence among each other.

#### 2.2.4 A new era

The cloning of NKCC2 and of its three main splice variants more than 25 years ago has allowed a great deal of knowledge to be gained on the functional characteristics, regulation, and physiological roles of this transport system along the TALH. It did so by providing the necessary information and tools to produce NKCC2‐ or variant‐specific probes, to express each of the variants in foreign cells as wildtype or mutated proteins, to generate NKCC2‐null ms models, and to link sequence variations in the encoding gene with specific traits or disorders in hu.

For reasons that have already been alluded to, the cell type exploited for the heterologous characterization of NKCC2 has consisted of the *X. laevis* oocyte almost exclusively. As will be shown later, it still played an important role in the progress attained. In the oocyte of *X. laevis*, moreover, foreign proteins commonly adopt their native functional characteristics and achieve high levels of activity (Long, O'Neill, & Cheeseman, 2018; Pike, Matthes, McSteen, & Gassmann, 2019; Wagner, Friedrich, Setiawan, Lang, & Broer, 2000).

### 2.3 Characterization of NKCC2 during the molecular era

#### 2.3.1 Localization of NKCC2 in animal species

##### Thick ascending loop of Henle

Localization of the NKCC2 variants in the kidney has relied solely on the detection of the encoding transcripts in the absence of available exons 4B‐, 4A‐ and 4F‐specific antibodies. In fact, these exons are probably not sufficiently immunogenic and divergent to allow for the production of sensitive and variant‐specific antibodies. Transcript‐based localization of the variants has still proved informative. It has also yielded comparable results among various studies and various species.

The renal distribution of the variants was assessed initially by Northern blot analyses of dissected rb kidney slices (Payne & Forbush, 1994) and microdissected rt TALH subsegments (T. Yang, Huang, Singh, Schnermann, & Briggs, 1996). These studies, which led to similar results, showed that NKCC2F was expressed in the medulla exclusively, NKCC2A in both the medulla and cortex and NKCC2B in the cortex exclusively. However, the microdissected tissues offered the advantage of demonstrating localization of NKCC2 in the TALH and of NKCC2B in the MD.

**Figure 4:**
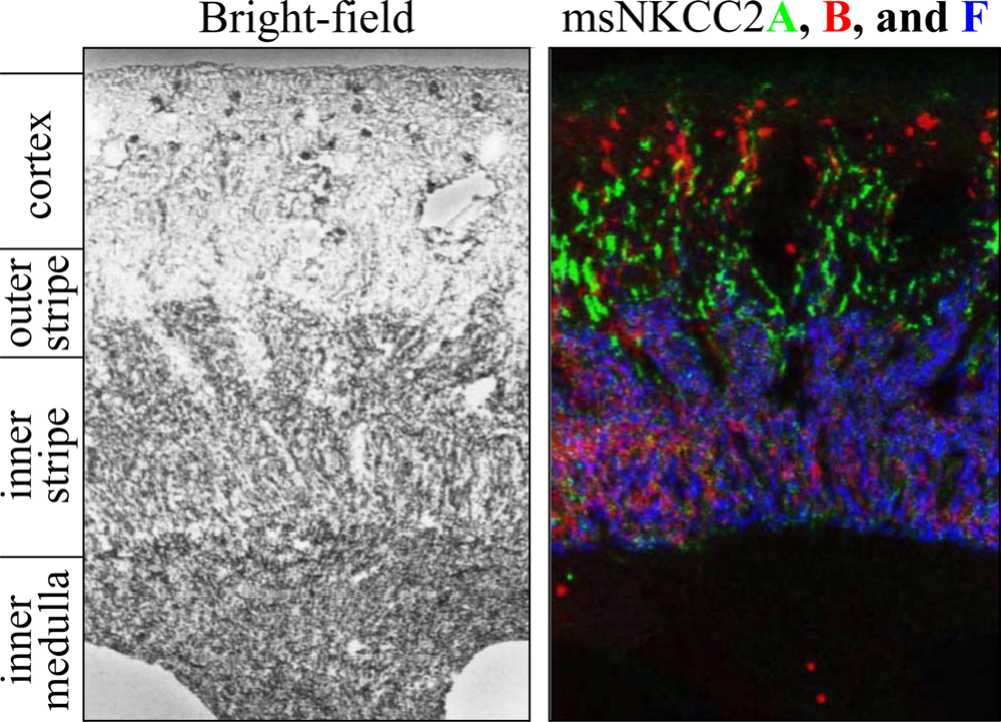
Distribution of Na^+^–K^+^–Cl^−^ cotransporter 2 (NKCC2) splice variants in adult ms kidney by in situ hybridization. Exon 4 was detected for each variant with a specific radioactive riboprobe on a sagittal tissue section, signals were converted to colors (red for NKCC2B, green for NKCC2A, and blue for NKCC2F) and images were superimposed. Adapted with permission from Igarashi et al. (1995). The color mount was generated by Dr Biff Forbush (Yale University, CT)

Distribution of the variants along the ms TALH was also assessed by in situ hybridization in two studies to obtain a better spatial resolution of the expression sites. The first study, which was by Igarashi, Vanden Heuvel, Payne, and Forbush (1995), showed that NKCC2F was expressed in the OMIS, NKCC2A in the outer medullary outer stripe (OMOS) and cortex, and NKCC2B in the periglomerular cortex (see Figure 4). The signal distribution also suggested the presence of both NKCC2B and NKCC2A in MD cells and the presence of all variants in both short and long nephrons. The second study, which was by Oppermann et al. (2006), showed similar results except that the localization of NKCC2B and NKCC2A in MD cells was more convincingly demonstrated.

As for the subcellular localization of NKCC2 along the TALH, it was assessed in rt by immunofluorescence (Kaplan et al., 1996) and ultrastructural studies (Nielsen et al., 1998). Interestingly, it was found that ∼5% of the carrier pool was expressed at the membrane and that the rest lied a large subapical reservoir. In these studies, NKCC2 was also identified in MD cells. The subcellular localization of NKCC2∆CT was also assessed in mice by immunofluorescence studies. Here, it was found that the truncated variant was present along the entire TALH but that it was expressed at the apical membrane in predominance (Mount, Baekgaard et al., 1999).

The abundance of each splice variant was also determined by Castrop and Schnermann (2008) in ms TALH through RNase protection assays using radiolabeled exon 4B‐, 4A‐, and 4F‐specific cRNA probes. The data obtained, which were only discussed in a review paper (Castrop & Schnermann, 2008), showed that the ratios of variant expression levels were ∼7NKCC2F:2NKCC2A:1NKCC2B. They were thus consistent with those of Igarashi et al. (1995) who had observed similar ratios in rb TALH by in situ hybridization (see Figure 4). However, the question of whether the same held true in both short and long nephrons does not appear to have been addressed in these studies or later on.

To date, the question of how the NKCC2 variants achieve differential localizations along the TALH remains unanswered. The mechanisms at play could involve exons B‐, A‐, and F‐specific pre‐messenger RNA sequence elements that can act as 3′ splice site enhancers upon their interaction with cell‐specific DNA‐binding proteins to prevent exon skipping. This hypothesis would imply that the exon‐specific inducible 3′ splice site enhancers are naturally silent or minimally active in the absence of such cofactors.

##### Other tissue

Just after its discovery, NKCC2 was said to be kidney‐specific based on the localization studies carried out (Gamba et al., 1994; Payne & Forbush, 1994). In the following years, however, it was found to exhibit a much wider distribution. This finding did not come as a surprise given that the detection of gene products has become progressively much more sensitive over the past 2 decades and that a large number of gene promoters appear to exhibit some degree of leakiness.

As of now, NKCC2 has been detected in ms pancreatic β‐cells (Alshahrani, Alvarez‐Leefmans, & Di Fulvio, 2012; Kelly et al., 2019), rt intestinal epitheliocytes and nervous plexus along a substantial portion of the gut (Xue et al., 2009), hu colonic epitheliocytes (Zhu, Xue, Ji, & Xing, 2011), rt, hu, and ms parietal and mucous gastric gland cells (T. Ji et al., 2012; Xue et al., 2009), and rt hypothalamic and neurohypophyseal cells (Konopacka et al., 2015). In the epithelial cell types where it was found, NKCC2 was generally localized on the apical side, and in ms pancreatic β‐cells, it consisted of the A variant. Through real‐time polymerase chain reaction studies, we have recently observed that NKCC2B and NKCC2A were also present in hu white blood cells (not shown).

On the basis of hu EST and RNA‐seq databanks^1^, ^2^, the distribution of the so‐called renal Na^+^–1K^+^–Cl^−^ cotransport appears to be even wider. These databanks show indeed that in addition to kidney, pancreas, intestine, and brain, NKCC2‐expressing tissues include (by alphabetical order) adrenal glands, bone, connective supports, eye, fat, liver, lung, muscle, parathyroid glands, spinal cord, testis, and vessels. In all of the extrarenal tissues listed, however, transcript abundance is much lower than in kidney with the notable exception of the pancreas. The hu protein atlas database^3^ further reveals that NKCC2 expression is upregulated in renal, liver, ovarian, and breast cancers.

Despite the wide distribution of NKCC2, the functional relevance of this isoform outside of the kidney has yet to be demonstrated or understood. In particular, the presence of functional Na^+^–K^+^–Cl^−^ cotransport uptake mechanisms on both the apical and basolateral membranes of the same epithelial cell would not call for an efficient arrangement to promote net vectorial transport of NaCl in one direction. For such an arrangement to make sense, one of the isoforms would have in fact to act as a low‐capacity housekeeping ion carrier system or the activity of the coexpressed isoforms would have to be differentially regulated. In the latter scenario, the orientation of transepithelial salt transport could vary as a function of the physiological needs to be met.

#### 2.3.2 Transport characteristics of NKCC2 in animal species

##### Stoichiometry

The variants have still not been characterized individually to confirm that they all couple the movement of ions in a stoichiometric rapport of 1Na^+^:1K^+^:2Cl^−^, harbor less or no more than four binding sites and are electrically silent. However, their transport dependence on extracellular [Na^+^], [K^+^], and [Cl^−^] was studied by different research groups and found to be consistent with a one ion‐binding site model for each cation and a two ion‐binding site model for Cl^−^ (Gagnon et al., 2004; Gagnon, Forbush, Caron, & Isenring, 2003; Gimenez & Forbush, 2007; Gimenez, Isenring, & Forbush, 2002). It should be nonetheless remembered that such models were inferred from Hill coefficients and that they thus only provide an estimate of the minimal number of binding sites involved (Garneau et al., 2017; Garneau, Marcoux et al., 2019; Garneau, Slimani et al., 2019; Marcoux et al., 2017).

There are in fact reasons to consider the possibility of alternate ion‐coupling models. As mentioned, for instance, renal tubule microperfusion studies have revealed the existence of a furosemide‐sensitive K^+^‐independent Na^+^–Cl^−^ cotransport mechanism in ms TALH (Sun et al., 1991). Along the same line, NKCC1 was shown to act as a 2Na^+^:1K^+^:3Cl^−^ cotransport system in certain cells such as squid neurons and ferret erythrocytes (Hall & Ellory, 1985; Russell, 1983). Lastly, we have found that the dependence of NKCC2A on extracellular [Cl^−^] in Cl^−^‐depleted *X. laevis* oocytes was best described by a model of anion binding at three or more sites (personal observations).

On the basis of transport assays in oocytes, the furosemide‐sensitive K^+^‐independent cotransport moiety that had been observed in ms TALH (Sun et al., 1991) was eventually attributed to the truncated NKCC2∆CT variants by a group of the investigator (Plata et al., 2001). These studies showed more specifically that when cAMP_i_ was reduced pharmacologically or when extracellular osmolality was changed from 200 to 100 mOsM, ^22^Na^+^ influx by NKCC2∆CT‐expressing oocytes was much higher compared to control oocytes even in the absence of extracellular K^+^. The NKCC2∆CT variants were thus considered to act as Na^+^–Cl^−^ cotransport systems under these circumstances.

Although interesting, the data of Plata et al. on the characteristics of NKCC2∆CT are difficult to interpret given that ^22^Na^+^ influx was actually seen to decrease importantly when extracellular osmolality was changed from 200 to 150 mOsM and that the kinetics of NKCC2∆CT activity as a function of [Na^+^], [K^+^ or Rb^+^], and [Cl^−^] were not reported. The data of Plata et al. are also difficult to interpret in the light of other studies. For instance, NKCC2∆CT was detected in ms TALH (Mount, Baekgaard et al., 1999) but not tested for its subcellular localization at low cAMP_i_ levels. In previous studies, the NKCC2 of LLCPK1 cells was also found to be K^+^‐dependent while no measures had been applied to alter cAMP_i_ (Brown & Murer, 1985).

The reported characteristics of NKCC2∆CT are also difficult to reconcile with known models of ion translocation by carrier systems. They imply that NKCC2 could sustain ion translocation even if its K^+^ transport site is no longer functionally active. Among various possibilities, NKCC2∆CT could do so if its K^+^ transport site was also able to translocate Na^+^ or if its translocation pocket was able to face the extracellular side even when it is not completely emptied (Lytle et al., 1998). These possibilities would still not explain how the absence of the distal C‐terminus would affect the properties of the ion‐binding sites or would elicit partial reactions during the transport cycle.

##### Kinetics of ion transport

The apparent ion affinity (*K*_m_) and maximal transport capacity (*V*_max_) of the NKCC2 splice variants have been determined by at least three research groups in the oocyte expression system (Gimenez et al., 2002; Marcoux et al., 2019a, 2019b; Plata, Meade, Vazquez, Hebert, & Gamba, 2002). Among the experiments carried out by these research groups, the NKCC2 analyzed were not all from the same species and the conditions used differed to some degree. Yet, each of the variants was found to share similar transport features. It is in fact between the variants themselves that most of the differences were observed.

**Figure 5:**
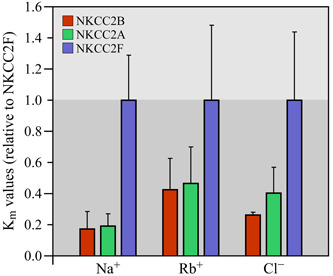
Apparent affinities of Na^+^–K^+^–Cl^−^ cotransporter 2 (NKCC2) splice variants for Na^+^, Rb^+^, and Cl^−^. The data shown correspond to mean *K*_m_ ratios ± standard error among three independent studies (as described in the text). In each study, these ratios were obtained by dividing the mean *K*_m_ determined for either variant with the mean *K*_m_ determined for NKCC2F. These constants are all seen to be higher for NKCC2F and analogous between NKCC2A and NKCC2B. On the basis of our observations, *V*_max_ was also found to be higher for NKCC2F and similar between NKCC2A and NKCC2B (not shown)

The data reported are reproduced in Figure 5 for the ion affinities as normalized *K*_m_ values to provide an outlook of how the variants differed among each other. In either study, normalized *K*_m(Na+)_, *K*_m(Rb+)_, or *K*_m(Cl−)_ are seen to be higher for NKCC2F (especially in the case of *K*_m(Na+)_) such that this variant appears to exhibit the lowest affinities for all three ions. In Figure 5, the normalized *K*_m_ values are also seen to be substantially lower for NKCC2B and NKCC2A but still quite similar between the two variants. As for the *V*_max_ values, they were not reported in either of the studies. From our own experiments (Marcoux et al., 2019b), however, they were found to be much higher for NKCC2F and relatively similar between the other two variants (data not published).

##### Residues involved in ion transport

Given that the main splice variants are identical to each other except for the residue sequence of the TMD2–CL1 domain, their individual functional characteristics have to be explained by the variable composition of exon 4 specifically. In this regard, and as can be seen in Figure 3, 10 out of 18 residues in TMD2 and 3 out of 11 residues in CL1 differ among the variants of rb and the same is true for those of many other animal species. However, differences in physicochemical properties among the nonconserved residues still translate into relatively low Grantham distances (∼80 or less). In shark, the TALH is devoid of NKCC2B but the sites and degree of residue divergence between NKCC2A and NKCC2F are still the same.

Mutagenic analyses did confirm the role of exon 4 as an affinity‐specifying domain and allowed to identify the sites at play. The first series of analyses were designed to study the effect of interchanging divergent residues between shark NKCC2A and NKCC2F on the ion‐dependence of ^86^Rb^+^ transport (Gagnon, Bergeron, Daigle, Lefoll, & Isenring, 2005; Gagnon et al., 2004). They showed that while the sites of functional relevance were localized in the middle portion of the TMD2–CL1 segment (Figures 1a and 3), those of TMD2 were key in specifying Na^+^, Rb^+^, and Cl^−^ affinity and those in CL1 in specifying Cl^−^ affinity for one of the binding sites. Another series of similar analyses were designed to study the effect of interchanging divergent residues between three rbNKCC2 variants. They led to analogous findings but further revealed that the kinetic characteristics of NKCC2B were similar to those of NKCC2A (Gimenez & Forbush, 2007).

#### 2.3.3 Structure of NKCC2

The predicted structural model of NKCC2 was initially found to be very similar to that of NKCC1 but not surprisingly so as both carriers share over 60% identity in residue sequence (Gamba et al., 1994; Igarashi et al., 1995; Payne & Forbush, 1994). Since then, these models have been confirmed for NKCC1 through a variety of indirect approaches and low‐resolution imaging studies (Gerelsaikhan & Turner, 2000; Jacoby, Gagnon, Caron, Chang, & Isenring, 1999; Monette, Somasekharan, & Forbush, 2014; Pedersen, Carmosino, & Forbush, 2008; Somasekharan, Monette, & Forbush, 2013; Somasekharan, Tanis, & Forbush, 2012). Both NKCC1 and NKCC2 were also found to assemble as homodimers (Moore‐Hoon & Turner, 2000; Parvin & Turner, 2011; Simard et al., 2007, 2004; Starremans et al., 2003) through the involvement at least four self‐interacting domains in their C‐termini (Brunet et al., 2005; Simard et al., 2004).

**Figure 6:**
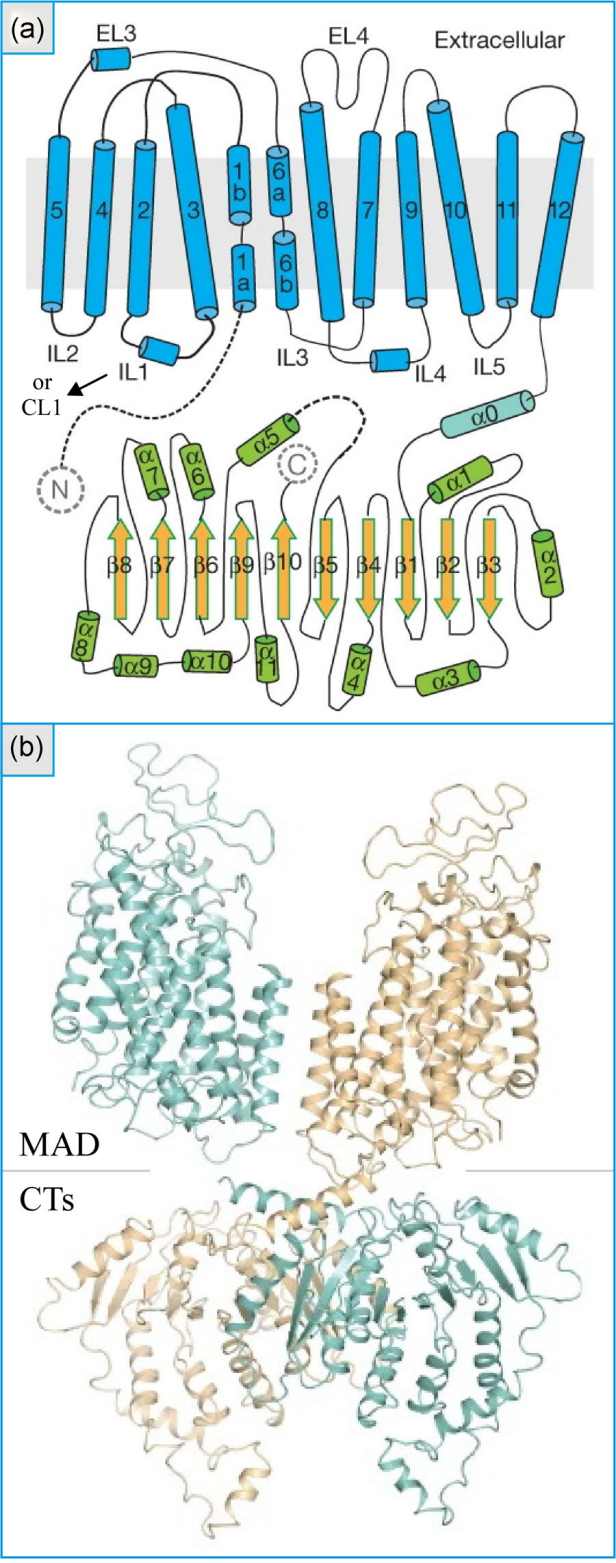
Structure of zebrafish Na^+^–K^+^–Cl^−^ cotransporter 1 based on a cryo‐electron microscopy density map. (a) Topology model of a monomer. Are shown: the transmembrane domains, extracellular loops (ELs), intracellular loops (ILs; also called connecting loops [CLs] in the text), linker helix (α0), as well as α‐ and β‐helices in the C‐terminus. (b) Structural model of a dimer. Are seen: the membrane‐associated domain (MAD) and the C‐termini (CTs). Adapted with permission from Xu et al. (1994)

More recently, Xu et al. (1994) have reported the first three‐dimensional cryo‐electron microscopy density map of zebrafish NKCC1 at a ∼3‐Å resolution (Figure 6). This map confirmed that NKCC1 was a 12‐TMD protein and that two of the formerly identified self‐interacting domains (corresponding to linker helix α0 after TMD12 and strand β3/helix α3 before the middle C‐terminus in Figure 6a) acted as dimerization contact points. The map was also unexpected to some extent (Garneau & Isenring, 2019). For instance, it showed that the membrane domains adopted an inverted repeat architecture arrangement in each monomer, interacted with the intracellular C‐terminus of the opposite monomer in a domain swap configuration, and took the form of a horseshoe in their joint state (Figure 6a,b). The density map suggested thus that the ion‐binding sites of each monomer were not sufficiently close to form a unique translocation pocket.

In simulation analyses Chew et al. further showed that the CL1 domain of one monomer could interact with the C‐terminus of the opposite monomer. This finding was of special interest in view of the role that the TMD2–CL1 domain of NKCC2 was found to play (Gagnon et al., 2004, 2005, 2002; Gimenez et al., 2002; Marcoux et al., 2019a, 2019b; Plata et al., 2002). Similar analyses also showed that the ion‐binding sites were contained within a solvent‐accessible vestibule that was delineated by TMD1, TMD3, TMD6, and TMD8. On the basis of these findings, it would thus appear that affinity specification is not coordinated by the ion‐binding sites themselves. Otherwise, evidence was also provided that ion binding by NKCC1 was cooperative and that each of the monomers harbored more than one Cl^−^ transport site.

#### 2.3.4 Regulation of NKCC2

NKCC2 is activated through phosphorylation of an S/T residue cluster (Figure 1a) in its N‐terminus (Gimenez & Forbush, 2005; Loureiro, Barros, Matos, & Jordan, 2019). Yet, a little is known regarding the mechanisms through which the addition of PO_4_^2‐^ groups to this cluster causes ion cotransport to increase. Among other possibilities, such mechanisms could involve a change in the conformation, abundance, or quaternary structure of NKCC2 at the cell surface. Whether the termini could harbor additional phosphoacceptor sites of importance is also possible (Loureiro et al., 2019).

On the basis of in vitro studies, oxidative stress‐responsive kinase 1 (OSR1) and STE20/SPS1‐related proline/alanine‐rich kinase (SPAK) are two of the enzymes that were found to phosphorylate NKCC2 at the N‐terminus (Patel & Gelman, 1980; Richardson et al., 2011). However, they must rely on other phosphorylating enzymes to acquire an active state (Ferdaus et al., 2016; Richardson & Alessi, 2008), on Ca‐binding protein 39 to assemble into a functional homodimer (Filippi et al., 2011; Mehellou, Alamri, Dhiani, & Kadri, 2018; Ponce‐Coria, Gagnon, & Delpire, 2012) and on the protein‐related receptor with A‐type repeats 1 to reach the membrane (Reiche et al., 2010). Note that SPAK could play a more minor role in vivo as it is expressed at much lower levels than OSR1 in TALH cells (Grimm et al., 2012).

Among the effectors that can phosphorylate NKCC2 directly, OSR1/SPAK have received most of the attention. However, there is evidence to suggest that NKCC2 can be N‐terminally phosphorylated by PKA at T_96_ and S_126_ in rt (Gunaratne et al., 2010), by SYK at Y_45_ in ms (Loureiro et al., 2019) and by AMPK at S_126_ in rb (Fraser et al., 2007) and that it can be C‐terminally phosphorylated by PKA at S_874_ in rt (Gunaratne et al., 2010). PP1 could also act on the phosphoacceptor sites of NKCC2 given that it has been found to play a key role in the regulation of other CCCs and that a relevant PP1 interacting site has been identified in the N‐terminus of NKCC1 (Darman, Flemmer, & Forbush, 2001).

The WNK kinases have received great attention as well but were found to act through phosphoactivation of OSR1/SPAK rather than on NKCC2 itself (Alessi et al., 2014; Richardson & Alessi, 2008; J. Zhang, Siew, Macartney, O'Shaughnessy, & Alessi, 2015). Their role as indirect effectors was suggested most convincingly by the observation that in the TALH of OSR1^+/−^ mice (where OSR1 activity is reduced) and of knockin SPAK^T243A/T243A^ mice (where SPAK cannot be activated by the WNK kinases), NKCC2 phosphorylation was impaired (Lin et al., 2011; Rafiqi et al., 2010). On the basis of localization or functional studies, however, only two of the WNK kinases (WNK1 and WNK3) are likely to play a role in NKCC2 regulation along the TALH (Cheng, Truong, Baum, & Huang, 2012; Ponce‐Coria et al., 2014, 2008; Rinehart et al., 2005).

Previous studies in isolated TALH have provided key insight into the mechanisms of NKCC2 activation by signaling enzymes. In particular, they revealed that NKCC2 could be recruited at the cell surface from subapical vesicles (Ares, 2019; Ares, Caceres, Alvarez‐Leefmans, & Ortiz, 2008; Caceres, Ares, & Ortiz, 2009; Caceres, Mendez, Haque, & Ortiz, 2016; Ortiz, 2006) and that it underwent phosphorylation under such circumstances (Gimenez & Forbush, 2003; Gunaratne et al., 2010). Even if these studies were conducted to understand the bases of NKCC2 regulation by cAMP or cGMP, they still pointed towards a key role for the trafficking machinery in response to carrier phosphorylation.

There are now new lines of evidence to indicate that the WNK–OSR1/SPAK pathway stimulates NKCC2‐mediated Na^+^–K^+^–Cl^−^ cotransport by acting on the trafficking machinery. Indeed, Marcoux et al. (2019b) have recently exploited the oocyte expression system to show that cell shrinkage increased NKCC2 expression to the membrane in the presence of WNK kinases. These results were in keeping with previous observations that SPAK regulates NKCC2 expression at the surface of TALH cells (Rafiqi et al., 2010; J. Zhang et al., 2015) and that it is activated during the hypertonic condition through phosphorylation of a Ser residue in its kinase domain (Vitari, Deak, Morrice, & Alessi, 2005; Zagorska et al., 2007).

Marcoux et al. (2019b) were also the first research group to show that the NKCC2 splice variants were differentially regulated by the WNK–OSR1/SPAK pathway and that the mechanisms of carrier upregulation varied as a function of the WNK at play. In the presence of WNK3, for instance, activation of this pathway increased cell surface expression of NKCC2A and NKCC2B much more than of NKCC2F. In the presence of WNK1, however, it increased ion cotransport by all of the variants but led to minimal changes in carrier expression or distribution (Marcoux et al., 2019b). These findings also suggested that exon 4 harbored specific residues that allow the CL1 domain to interact with a component of the trafficking machinery.

Through subsequent studies, a mutagenic approach allowed Marcoux et al. (2019a) to identify variants residues in CL1 (at Positions 230 and 238) that endowed each of the variants with unique trafficking and ion transport characteristics. From these studies, it was concluded that CL1 could be part of a key regulatory motif that was not only involved in carrier trafficking but that also contributed to the differential properties of Na^+^–K^+^–Cl^−^ cotransport along the TALH. From a broader perspective, it was furthermore concluded that the N‐terminus of NKCC2 could play a role in carrier trafficking by regulating the access of CL1 to the trafficking machinery.

There are additional lines of evidence to suggest that the trafficking machinery plays an important role in NKCC2 regulation by various hormones. While, for instance, aldosterone has been shown to downregulate NKCC2 expression in the TALH as part of the so‐called escape phenomenon (Madala Halagappa, Tiwari, Riazi, Hu, & Ecelbarger, 2008; Turban, Wang, & Knepper, 2003) and to exhibit purinergic‐dependent activity (Y. Zhang, Listhrop, Ecelbarger, & Kishore, 2011), both this hormone and purinergic agonists have also been shown to affect the abundance of other ion transport systems at the surface of the renal epithelium (Raghavan & Weisz, 2015; Ware, Rasulov, Cheung, Lott, & McDonald, 2020). In this regard, additionally, aldosterone has been found to increase phosphorylation and membrane abundance of NCC (van der Lubbe et al., 2011) and to coordinate WNK activity in several nephron segments (Susa et al., 2012).

#### 2.3.5 Physiological roles of NKCC2

##### Primary roles of the variants along the TALH

NKCC2F is expressed in the OMIS portion of the TALH, is approximately twice as abundant as the other variants and is a low‐affinity high‐capacity carrier (Gagnon et al., 2003; Gimenez & Forbush, 2007; Gimenez et al., 2002; Igarashi et al., 1995; Itoh et al., 2014; Marcoux et al., 2019b; Plata et al., 2002). In a nephron segment where the luminal salt concentration is very high, NKCC2F is thus well adapted to ensure high levels of NaCl reabsorption (Marcoux et al., 2019b). Its importance in this matter is supported by the observations that NKCC2‐null mice die soon after the birth of severe polyuria (Takahashi et al., 2000) while NKCC2B‐ or NKCC2A‐null mice exhibit mild phenotypes (Oppermann et al., 2006, 2007).

As for NKCC2A, it is expressed in the OMOS, cortical, and MD portions of the TALH, is approximately twice as abundant as NKCC2B, and is a high‐affinity low‐capacity carrier (Carota et al., 2010; Gimenez et al., 2002; Igarashi et al., 1995; Itoh et al., 2014; Marcoux et al., 2019b). In a nephron segment where luminal salt concentration has decreased from the activity of NKCC2F and will further decline up to MD cells, NKCC2A is thus well suited to ensure ion reabsorption along all of the cortical TALH (Ares, Caceres, & Ortiz, 2011; Castrop & Schnermann, 2008) and prevent excessive NaCl delivery to the MD, a structure where solute reabsorption must be fine‐tuned (Ares et al., 2011; Bailly, 2000; Maack, 1996). Given, moreover, that the cortical collecting duct ensures higher levels of water reabsorption than the medullary collecting duct in response to vasopressin (Itoh et al., 2014), NKCC2A could also play a particularly important role during antidiuretic states.

A question that comes to mind is whether NKCC2A could have been substituted for by NKCC2F along the TALH. If it had, that is, if its affinity for Na^+^ and Cl^−^ ions had been much lower compared to that of NKCC2B, salt sensing by NKCC2F‐expressing MD cells would have probably not been as sensitive at the lower range of ion concentration (Carota et al., 2010; Itoh et al., 2014; Orlov & Mongin, 2007). Even if its transport capacity is not as high as NKCC2F, importantly, NKCC2A is still predicted to operate at near‐saturation along the entire cortical TALH such that it has the potential of acting as an efficient carrier system from OMOS to periglomerular cortex.

Intriguingly, Oppermann et al. (2006, 2007) found that NKCC2A‐null mice exhibited no more than a mild decrease in steady‐state urine osmolality and concentrating ability. At the same time, these mice also expressed higher levels of NKCC2B in the cortex and probably developed a less striking phenotype as a consequence. Cl^−^ absorption by their TALH was still impaired based on microperfusion studies under high‐flow conditions, confirming that NKCC2A probably does not play a redundant role. In and of themselves, however, these studies did not inform on the transport capacity of NKCC2A as NKCC2B probably allowed Cl^−^ to be fully reabsorbed under low‐flow conditions.

As for NKCC2B, it is expressed in the late cortical TALH, MD included and is also a high‐affinity low‐capacity carrier (Carota et al., 2010; Gimenez et al., 2002; Igarashi et al., 1995; Itoh et al., 2014; Marcoux et al., 2019b). In a nephron segment where the luminal salt concentration is maximally reduced, NKCC2B should thus be able still to ensure net ion retrieval. At the same time, it would unlikely act as a sensitive salt sensor by MD cells given that its activity saturates far below the luminal concentration of Na^+^ and Cl^−^ ions in the distal TALH (Orlov & Mongin, 2007). There is also the possibility that the in vivo kinetic traits of NKCC2B differ from those reported in oocytes.

An NKCC2B‐null ms model was also characterized by Oppermann et al. (2006) and found to exhibit a mild decrease in urine concentrating ability. In this model, however, the ablated variant was not compensated for by NKCC2A and NKCC2F, confirming that the NKCC2A‐null model would have probably exhibited a more severe phenotype in the absence of compensation by NKCC2B (Oppermann et al., 2007). Microperfusion studies further showed that Cl^−^ absorption along the NKCC2B‐null TALH was decreased once again but under low‐flow condition only, thereby implying that NKCC2A is not a high‐capacity carrier system either.

To study more specifically the role of NKCC2 in TGF, Oppermann et al. (2006, 2007) used their ms models to carry out proximal tubular flow measurements in response to TALH perfusion and serum renin measurements in response to intravenous saline infusion. The first approach showed that TGF was markedly altered in both models but under the low‐flow conditions in NKCC2B‐null mice and high‐flow conditions in NKCC2A‐null mice. As for the second approach, it showed (somewhat surprisingly) that renin suppression was only blunted in the NKCC2A‐null mice. However, it should be kept in mind that differences in compensatory expression of the nonablated variants could have accounted for these divergent responses and increased even further over time.

They are still uncertainties as to how NKCC2 could contribute to TGF. On the basis of previous work, the variants at work could act by inducing changes in the release of vasoactive substances by juxtaglomerular cells through changes in MD volume such as occur in the case of renin (Gonzalez, Salomonsson, Muller‐Suur, & Persson, 1988; Hanner et al., 2008; Komlosi, Fintha, & Bell, 2006; Peti‐Peterdi, Morishima, Bell, & Okada, 2002). According to this possibility, higher concentrations of Na^+^ and Cl^−^ in the distal TALH would cause MD cells to swell (through increased NaCl uptake by NKCC2) and renin release to decrease, while NKCC2A or NKCC2B inhibition by furosemide would cause MD cells to shrink (through decreased NaCl uptake) and renin release to increase.

As for the NKCC2AF and NKCC2∆CT variants, their potential roles have already been discussed to some extent. Although they have been found to exert dominant‐negative effects on other NKCC2 variants, there is still little evidence to suggest that they could affect TALH function under normal, adaptive, or pathological conditions. Be that as it may, it is noteworthy that while NKCC2AF is expressed at high levels in shark kidney, it is not transcriptionally poised in other species under all circumstances (see subsection below).

##### Secondary roles along the TALH and systemic repercussions

**Figure 7:**
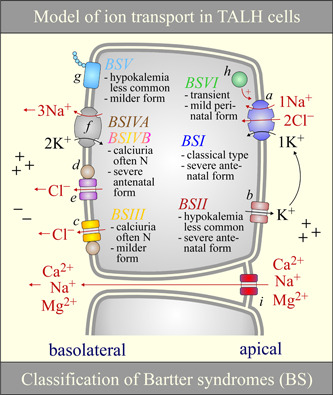
Model of ion transport and causes of NKCC2 dysfunction in the TALH. Ion transport: K^+^ recycling through K^+^ channels (b) in the apical TALH and through K^+^ channels (not shown) and the Na^+^/K^+^ ATPase pump (f) in the basolateral TALH cause both the luminal and serosal fluid to be positively charged (Greger, 1985; Greger & Schlatter, 1981; Hamilton & Devor, 2012; Hebert & Andreoli, 1984; Hebert, Culpepper, & Andreoli, 1981; Hurst, Duplain, & Lapointe, 1992). However, outwardly directed Cl^−^ conductive pathways (c–e) in the basolateral TALH decreases the effect of K^+^ recycling on the serosal side such that active NaCl reabsorption in this nephron segment causes the luminal‐to‐serosal transepithelial potential to increase along with the paracellular reabsorption of certain cations (Gu et al., 2009; Guinamard, Chraibi, & Teulon, 1995; Winters, Zimniak, Mikhailova, Reeves, & Andreoli, 2000; Yoshitomi, Koseki, Taniguchi, & Imai, 1987). NKCC2 dysfunction: Different types of Bartter syndromes (BS) are listed along with an overview of their clinical manifestations (see Cunha and Heilberg (2018) as to why these manifestations vary among the types). The color of the headings “BS” match those of the proteins affected. BSIVB appears in various shades given that it is associated with pathogenic mutations in both CLCNKA and CLCNKB. In this figure, the salt‐losing nephropathy that has been linked to melanoma antigen D2 has term BSVI arbitrarily. The proteins shown are (a) NKCC2 (SLC12A1), (b) ROMK2 or ROMK3 (KCNJ1), (c) CLCNKB, (d) Barttin, (e) CLCNKA, (f) the Na^+^/K^+^ ATPase, (g) Ca^2+^‐sensing receptor, (h) melanoma antigen D2, and (i) claudins (isoforms 14, 16, or 19). N, normal; NKCC2, Na^+^–K^+^–Cl^−^ cotransporter 2; TALH, thick ascending loop of Henle

As partly illustrated through Figure 7, NKCC2 plays many of its systemic roles by allowing or driving the reabsorption of Na^+^, Cl^−^, Ca^2+^, and Mg^2+^ by the TALH, the excretion of K^+^ and H^+^ by the initial collecting duct and the reabsorption of water throughout the collecting duct (Carmosino et al., 2015; Di Stefano, Roinel, de Rouffignac, & Wittner, 1993; Quamme, 1989; Simon et al., 1996; Xu et al., 1994). The importance of Na^+^–K^+^–Cl^−^ cotransport in these processes is illustrated by the potential of loop diuretics to cause volume contraction, water disorders (by lowering the urinary osmolar range), hypomagnesemia, hypokalemia, and metabolic alkalosis.

NKCC2 plays many other important systemic roles. Through its ability to act as a Na^+^–NH_4_^+^–Cl^−^ cotransport mechanism, it prevents NH_4_^+^ from building up in the general circulation during states of enhanced renal ammoniogenesis (as occur in acidemic states) and increases the buffering power of urine by driving the delivery of NH_3_ to the lumen of the collecting duct (Weiner & Verlander, 2011). Through its involvement in the TGF mechanism, along the same line, it regulates the release of renin and of angiotensin II from the juxtaglomerular apparatus to other tissues including the adrenal glands and resistive arteries (Oppermann et al., 2006, 2007).

The importance of NKCC2 in ECVF maintenance, water homeostasis, and acid‐base balance is suggested further by the observation that NKCC2 expression increases in response to a low‐salt diet, water restriction, or acidosis and decreases in response to high‐salt diets or water loading (Hao, Hao, & Ferreri, 2018; Hao, Salzo, Hao, & Ferreri, 2020; Karim, Attmane‐Elakeb, Sibella, & Bichara, 2003; L. Yang et al., 2018). In this regard, however, Brunet et al. (2005) have observed that expression of the NKCC2 variants along the ms TALH was differentially altered in response to water loading (↓F; ↔A; ↓AF) and furosemide administration (↑A; ↓F; ↓AF). In a subsequent study, another group of investigators (Schiessl et al., 2013) came to similar conclusions. Taken together, these findings are once more consistent with the idea of regulatable cell‐specific proteins that act along the TALH on inducible 3′ splice site enhancers in exon 4.

Genotype–phenotype correlation studies have also pointed to the key contribution of NKCC2 in BP control. For instance, one study showed that NKCC2A‐to‐NKCC2F expression ratios were higher in salt‐sensitive Dahl kidney than they were in salt‐resistant Dhal kidney (Herrera, Lopez, & Ruiz‐Opazo, 2001). Given that such ratios should be inversely proportional to NaCl reabsorption by the TALH, the NKCC2 variants in these models could have adapted to salt sensitivity rather than have been the cause of it. Another study showed that loss‐of‐function variants in *Nkcc2* were associated with lower BPs in a subcohort of 2,492 subjects from the Framingham Heart Study (Acuna et al., 2011; W. Ji et al., 2008; Monette, Rinehart, Lifton, & Forbush, 2011; Nandakumar, Morrison, Grove, Boerwinkle, & Chakravarti, 2018) and gain‐of‐function variants in *UMOD* with an NKCC2‐mediated increase in salt sensitivity (Graham et al., 2014; Mutig et al., 2011; Trudu et al., 2013).

#### 2.3.6 Hereditary forms of NKCC2 dysfunction

As mentioned earlier, the activity of NKCC2 is affected by that of many ion transport systems along the TALH. In particular, it is stimulated under conditions where luminal‐to‐cytosol [Na^+^], [K^+^], and [Cl^−^] gradients are increased. As such, a decrease in luminal K^+^ secretion (through primary ROMK inactivation), an increase in [Cl^−^]_i_ (through primary CLCNKB or BSND inactivation), and an increase in [Na^+^]_i_ (through primary Na^+^/K^+^‐ATPase pump inactivation) should all lead to a decrease in ion cotransport. Should also be associated with lower levels of NKCC2 activity, a defect in any of the factors that allow these transport systems to be expressed or conformationally active at the cell surface.

In light of such considerations, NKCC2 dysfunction could thus arise from a number of different anomalies along the TALH. In our opinion, and as laid out in Table 1, this type of dysfunction could be usefully designated as either primary, secondary, or tertiary, that is, under three etiologic groups where the anomalies are in the carrier itself, in other ion transport systems, or in regulatory factors, respectively. The various clinical presentations associated with a dysfunction of NKCC2 are also summarized in Figure 7.

**Table 1:** Classification of Bartter syndromes

NKCC2 dysfunction	Genes linked to phenotype	Mechanisms of abnormal renal salt handling along the TALH
Primary	NKCC2 (SLC12A1)	Decreased Na^+^–K^+^–Cl^−^ uptake via apical membrane^a^
Secondary	ROMK (KCNJ1)	Decreased K^+^ secretion via apical membrane^b^
	CLCNKB	Decreased Cl^−^ absorption via basolateral membrane^c^
	Barttin (BSND)	Decreased Cl^−^ absorption via basolateral membrane^d^
	CLCNKA+B	Decreased Cl^−^ absorption via basolateral membrane^e^
	Na^+^/K^+^ ATPase	Decreased Na^+^ absorption from lack of ATP^f^
Tertiary	CaSR	Decreased expression/activity of ROMK^g^
	MAGED2	Decreased expression of NKCC2^h^
	UMOD	Decreased expression of NKCC2^i^

*Note*: At least nine different defects have been associated with the hereditary forms of Bartter syndrome. They are classified here into primary NKCC2 dysfunction, where the defect is in NKCC2 itself, secondary NKCC2 dysfunction, where the defect is in another NKCC2‐dependent ion transport system, and tertiary NKCC2 dysfunction, where the defect is in a protein that regulates NKCC2 expression.

Abbreviations: CaSR, calcium‐sensing receptor; MAGED2, melanoma‐associated antigen D2; NKCC2, Na^+^–K^+^–Cl^−^ cotransporter 2; TALH, thick ascending loop of Henle; UMOD, uromodulin (also known as Tamm–Horsfall protein).

a Simon et al. (1996); Starremans et al. (2003); Vargas‐Poussou et al. (1998).

b Finer et al. (2003); Peters et al. (2002); Simon et al. (1996).

c Birkenhager et al. (2001); Kramer, Bergler, Stoelcker, and Waldegger (2008); Schlingmann et al. (2004); Scholl et al. (2006); Simon et al. (1997).

d Birkenhager et al. (2001); Kramer et al. (2008); Schlingmann et al. (2004); Scholl et al. (2006); Simon et al. (1997).

e Birkenhager et al. (2001); Kramer et al. (2008); Schlingmann et al. (2004); Scholl et al. (2006).

f Dimke, Hoenderop, and Bindels (2009); Finsterer and Scorza (2017).

g Gamba and Friedman (2009); Hebert, Brown, and Harris (1997); Watanabe et al. (2002).

h Kleta and Bockenhauer (2018); Laghmani et al. (2016).

i Mutig et al. (2011); Renigunta et al. (2011); Trudu et al. (2013).

## 3 CONCLUSION AND PERSPECTIVES

In this review, we have seen that NKCC2 is of crucial importance to the normal operation of the TALH as it contributes to extracellular fluid volume maintenance, urinary concentration/dilution, TGF, Mg^2+^ homeostasis, and acid‐base balance. We also provided new insights and hypotheses on the purposes of each variant, the functional characteristics and structural determinants of ion translocation by these carriers, and the mechanisms through which ion cotransport is regulated.

A remaining challenge in the field NKCC2‐mediated ion cotransport is the obtaining of protein probes and inhibitors that are specific to each of the variants. A recent publication on the high‐resolution structure of NKCC1 could become quite useful to this end by aiding in the design of such ligands. While the probes and inhibitors required could consist of pharmacologic agents or small peptides, prey sites would probably have to be included in the TMD2–CL1 domains.

The various clinical conditions that could benefit from the use of variant‐specific inhibitors include salt‐sensitive and glomerular hypertension (by targeting NKCC2F), inappropriate antidiuretic hormone secretion (by targeting NKCC2A), and excessive TGF response in the setting of acute tubular injury (by targeting NKCC2B). By contrast to loop diuretics, NKCC2F and NKCC2A inhibitors would also offer the advantage of preserving TGF and anti‐NKCC2B inhibitors of minimizing natriuresis.

Since it was cloned in the mid‐1990s, NKCC2 has been the object of ∼20 publications per year and has thus elicited continued interest over a long period of time. It will soon find a place of honor among the various ion transport systems of the TALH as further insight on the structural determinants of Na^+^–K^+^–Cl^−^ cotransport will be gained in the near future. It would be especially enlightening to study these determinants while the activity or phosphorylation of NKCC2 is altered through various maneuvers, drugs, or mutagenesis.

## CONFLICT OF INTERESTS

The authors declare that there are no conflict of interests.

## AUTHOR CONTRIBUTIONS

Conception, design, and drafting: A. A. M., A. P. G., and P. I. Critical revising: A. A. M., F. M.‐W., A. P. G., and P. I. All data authors contributed in acquisition, analysis, and interpretation.

## ETHICS STATEMENT

All persons designated as authors qualify for authorship and have approved the final version of the manuscript.

## ENDNOTES

1
https://www.ncbi.nlm.nih.gov/UniGene/ESTProfileViewer.cgi?uglist=Hs.123116.

2
https://www.ebi.ac.uk/gxa/home (query SLC12A1).

3
https://www.proteinatlas.org/ENSG00000074803-SLC12A1/pathology.
